# Association between frailty, delirium, and mortality in older critically ill patients: a binational registry study

**DOI:** 10.1186/s13613-022-01080-y

**Published:** 2022-11-17

**Authors:** Berhe W. Sahle, David Pilcher, Edward Litton, Richard Ofori-Asenso, Karlheinz Peter, James McFadyen, Tracey Bucknall

**Affiliations:** 1 School of Nursing and Midwifery, Faculty of Health, Deakin University, 221 Burwood Highway, Burwood, Melbourne, VIC 3125 Australia; 2 Centre for Quality and Patient Safety Research, Alfred Health Partnership, Institute for Health Transformation, Melbourne, VIC Australia; 3 Department of Intensive Care, Alfred Hospital, Melbourne, VIC Australia; 4 School of Public Health and Preventive Medicine, Monash University, Melbourne, VIC Australia; 5 Australian and New Zealand Intensive Care Society Centre for Outcome and Resource Evaluation, Melbourne, VIC Australia; 6 Fiona Stanley Hospital, Perth, WA Australia; 7 The University of Western Australia, Perth, WA Australia; 8 Department of Epidemiology and Preventive Medicine, Monash University, Melbourne, Australia; 9 Atherothrombosis and Vascular Biology, Baker Heart and Diabetes Institute, Melbourne, VIC Australia; 10 Department of Medicine, Central Clinical School, Monash University, Melbourne, VIC Australia; 11 Baker Department of Cardiometabolic Health, University of Melbourne, Melbourne, VIC Australia; 12 Department of Cardiology, The Alfred Hospital, Melbourne, VIC Australia; 13 Department of Clinical Hematology, The Alfred Hospital, Melbourne, VIC Australia

**Keywords:** Frailty, Delirium, Mortality, Critically ill, Intensive care unit, Length of stay, Clinical decision-making

## Abstract

**Background:**

Frailty and delirium are prevalent among older adults admitted to the intensive care unit (ICU) and associated with adverse outcomes; however, their relationships have not been extensively explored. This study examined the association between frailty and mortality and length of hospital stay (LOS) in ICU patients, and whether the associations are mediated or modified by an episode of delirium.

**Methods:**

Retrospective analysis of data from the Australian New Zealand Intensive Care Society Adult Patient Database. A total of 149,320 patients aged 65 years or older admitted to 203 participating ICUs between 1 January 2017 and 31 December 2020 who had data for frailty and delirium were included in the analysis.

**Results:**

A total of 41,719 (27.9%) older ICU patients were frail on admission, and 9,179 patients (6.1%) developed delirium during ICU admission. Frail patients had significantly higher odds of in-hospital mortality (OR: 2.15, 95% CI 2.05–2.25), episodes of delirium (OR: 1.86, 95% CI 1.77–1.95), and longer LOS (log-transformed mean difference (MD): 0.24, 95% CI 0.23–0.25). Acute delirium was associated with 32% increased odds of in-hospital mortality (OR: 1.32, 95% CI 1.23–1.43) and longer LOS (MD: 0.54, 95% CI 0.50–0.54). The odds ratios (95% CI) for in-hospital mortality were 1.37 (1.23–1.52), 2.14 (2.04–2.24) and 2.77 (2.51–3.05) for non-frail who developed delirium, frail without delirium, and frail and developed delirium during ICU admission, respectively. There was very small but statistically significant effect of frailty on in-hospital mortality (*b* for indirect effect: 0.00037, *P* < 0.001) and LOS (*b* for indirect effect: 0.019, *P* < 0.001) mediated through delirium.

**Conclusion:**

Both frailty and delirium independently increase the risk of in-hospital mortality and LOS. Acute delirium is more common in frail patients; however, it does not mediate or modify a clinically meaningful amount of the association between frailty and in-hospital mortality and LOS.

**Supplementary Information:**

The online version contains supplementary material available at 10.1186/s13613-022-01080-y.

## Background

Frailty is a multidimensional geriatric syndrome characterized by increased vulnerability to a range of adverse outcomes due to loss of physiological reserve [1]. The prevalence of frailty increases with age, and is associated with poorer health outcomes, including mortality [2], longer hospitalization [3] and increased health care costs [4]. The prevalence of frailty among people aged 65 years or over is 10%, increasing to 26% in people aged 85 years or over [5].

Delirium is a clinical syndrome characterized by inattention and global cognitive dysfunction, with prevalence rates ranging from 14 to 24% among hospitalized older adults, and 45 to 87% among patients admitted to an intensive care unit (ICU) [6, 7]. Delirium can result from diverse and multiple etiologies, and is associated with a higher risk of mortality, longer length of stay in ICU, more complications and long-term cognitive dysfunction [7, 8].

Evidence suggests that both frailty and delirium result from disintegration of balance and homeostasis across multiple body systems, and share multiple pathophysiologic pathways, such as inflammation, atherosclerosis, and nutritional deficiency [9]. Both delirium and frailty are also common among older adults and have multifactorial etiologies [10, 11]. It has been established that both frailty and delirium are independently associated with poor health outcomes [10, 12]. Furthermore, frailty is associated with a 2- to 6-fold higher risk of delirium [13, 14] which may influence the associations between frailty and mortality.

Despite existing evidence indicating that frailty and delirium increase the risk of adverse clinical outcomes, the complex relationship between frailty and delirium, and its impact on clinical outcomes has not been extensively explored. Whether the association between frailty and mortality is modified by acute delirium is unclear. In a prospective study of 977 adult ICU patients, frail patients who developed delirium during an ICU admission had a fourfold increased risk of death compared to non-frail patients who developed delirium in the ICU [13]. However, it has also been reported that delirium confers greater risk of mortality at lower levels of frailty [15]. Furthermore, considering that frailty is associated with higher risk of delirium, the effect of frailty on mortality may be partly through increased risk of delirium. To date, no previous studies have examined whether the association between frailty and mortality is mediated by acute delirium.

Using data from a large multicentre cohort of critically ill patients aged 65 and older, we examined associations of frailty with in-hospital mortality and length of stay, and whether these associations are modified or mediated by an episode of acute delirium.

## Methods

### Data sources and participants

We analysed data from the Australian and New Zealand Intensive Care Society (ANZICS) Adult Patient Database (APD), a binational clinical quality registry dataset run by the ANZICS Centre for Outcome and Resource Evaluation (CORE). Details of the ANZICS APD design has been published previously [16]. In brief, the ANZICS APD contains data from over 3 million patient episodes collected from 221 ICUs, representing 97% of Australia ICUs and 67% of New Zealand ICUs. The ANZICS CORE participating ICUs contribute de-identified data. Each contributing ICU allows subsequent data use as appropriate, understanding procedures and in compliance with the ANZICS CORE terms of reference.

### Assessment of frailty

Frailty was assessed using the modified version of the Canadian Study of Health and Aging Clinical Frailty Scale (CFS), a nine-point categorical scale judgement-based global assessment of fitness or degree of frailty. The CFS has been found to be valid and reliable for assessing frailty in acute care and community settings, including critically ill patients [17]. Consistent with previous studies, patients with CFS score of 5 or more were considered as frail [18]. Since 2017, frailty has been collected on admission to the ICU, depending on the patient’s level of physical function in the 2 months preceding admission. Frailty scores were assessed from clinical record by data collectors who did not receive any specific training on the use of CFS [18].

### Assessment of delirium

Acute delirium was diagnosed by the treating physician using standardized assessment tools such as (but not limited to) the Confusion Assessment Method for ICU (CAM-ICU) [19]. The CAM-ICU is one of the most frequently employed tools developed and validated to assess delirium in ICU patients [20]. Patients who develop delirium after discharge from ICU, were admitted to ICU due to delirium or with another diagnosis and are noted to have delirium present at the time of ICU admission, are excluded from delirium assessment. We included all critically ill patients aged 65 or older admitted to the ICU between 1 January 2007 and 31 December 2020.

The analyses were adjusted for risk of hospital mortality estimated using the Australian and New Zealand Risk of Death (ANZROD) model. The ANZROD model has excellent discrimination and good calibration and risk-adjustment for local case-mix variation [21]. ANZROD is derived from patient and clinical characteristics, including the Acute Physiology and Chronic Health Evaluation (APACHE) III, ICU admission source, admission diagnoses, Acute Physiology score, APACHE II and III chronic health score components, treatment limitation, and ventilation [22].

### Data analysis

We used descriptive statistics to summarize patient characteristics. We used mixed effects logistic regression models to assess the associations between frailty at admission, acute delirium, and in-hospital mortality and LOS. We investigated the mediation effect of delirium on the association between frailty and in-hospital mortality and LOS, using a Stata Macro for multilevel mediation analysis developed by Krull and MacKinnon [23]. To account for the right skewed distribution of LOS data, we log-transformed data on LOS to normality before analysis [24]. A bootstrap analysis (500 samples generated using nonparametric method) was used to test the significance of indirect effect in mediation analysis [25]. We also examined whether acute delirium modified the associations between frailty and mortality and LOS by fitting an interaction term of delirium and frailty in the models. *P* < 0.05 was considered significant in 2-sided tests (see Additional file 1).

## Results

**Fig. 1:**
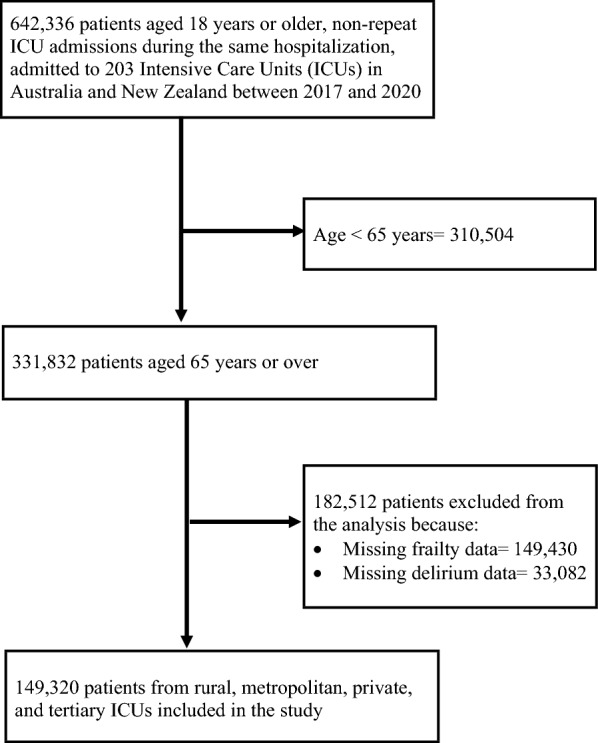
Inclusion and exclusion criteria and number of patients

In all, 149,320 patients aged 65 years or over (57.7% males) who had data on frailty and delirium were included in the analysis (Fig. 1). The median age of the included patients was 75.2 years (interquartile range [IQR], 70.3–81.1 years), and were admitted to 203 ICUs during the study period. More than one-fourth of the patients (27.9%) were classified as frail, and median frailty score was 4 (IQR, 3–5). Patient characteristics by acute delirium status are presented in Table 1. Overall, patient characteristics were comparable between those with and without missing data on frailty and delirium (Additional file 2: Table S1).

**Table 1:** Characteristics of the patients included in the analysis (*n* = 149,320)

Characteristics	Episode of acute delirium	No delirium	All patients
Number	9179 (6.1)	140,141 (93.9)	149,320
Age (years), median (IQR)	77 (71.5–82.8)	75.1 (70.3–81.0)	75.2 (70.3–81.1)
Sex (men)	5674 (61.8)	80,479 (57.4)	86,153 (57.7)
Frail, yes	4142 (45.1)	37,577 (26.8)	41,719 (27.9)
Frailty score, median (IQR)	4 (3–6)	4 (3–5)	4 (3–5)
Admission diagnosis			
Cardiovascular disease	2270 (24.7)	38,456 (27.4)	40,726 (27.3)
Gastrointestinal	1432 (15.6)	25,503 (18.2)	26,935 (18.0)
Respiratory disease	1315 (14.3)	20,866 (14.9)	22,181 (14.8)
Sepsis	1413 (15.4)	11,939 (8.5)	13,352 (8.9)
Neurological disorders	735 (8.0)	11,909 (8.5)	12,644 (8.5)
Chronic respiratory disease	1215 (13.2)	15,653 (11.2)	16,868 (11.3)
Cardiovascular disease	1612 (17.6)	21, 883 (15.6)	23,493 (15.7)
Chronic renal failure	578 (6.3)	6784 (4.8)	7362 (4.9)
Chronic liver disease	189 (2.1)	1373 (0.9)	1562 (1.0)
Immunosuppressive disease	282 (3.1)	3032 (2.2)	3314 (2.2)
Immunosuppressive therapy	562 (6.1)	7130 (5.1)	7692 (5.1)
Metastatic cancer	385 (4.2)	6967 (5.0)	7352 (4.9)
Hospital type			
Tertiary	3734 (40.7)	37,282 (26.6)	41,016 (27.5)
Metropolitan	1888 (20.6)	23,697 (16.9)	25,585 (17.1)
Rural/regional	1874 (20.4)	23,334 (16.6)	25,208 (16.9)
Private	1683 (18.3)	55,828 (39.8)	57,511 (38.5)
Planned admissions to ICU after elective surgery	1902 (20.7)	60,775 (43.4)	62,677 (42.0)
APACHE III score, median (IQR), %	67 (55–82)	53 (43–66)	54 (43–67)
APACHE III predicted mortality, mean (SD), %	25.0 (22.2)	14.3 (18.6)	15.0 (19.0)
ANZROD, median (IQR), %	8.7 (2.6–23.9)	2.1 (0.7–8.2)	2.3 (0.7–9.1)
ANZROD, mean (SD), %	17.2 (20.7)	9.0 (16.7)	9.5 (17.1)
ICU admission source			
Operating theatre	3638 (39.6)	82,547 (58.9)	86,185 (57.7)
Emergency department	2846 (31.0)	31,497 (22.5)	34,343 (23.0)
Hospital ward	1993 (21.7)	19,754 (14.1)	21,747 (14.5)
Direct transfer from other ICU	205 (2.2)	1277 (0.9)	1482 (1.0)
Direct admission from other hospital	472 (5.1)	4560 (3.2)	5032 (3.4)
Direct admission from home	10 (0.1)	405 (0.3)	415 (0.3)
Length of hospital stay, median (IQR), d	14.1 (8.2–25.0)	8.2 (4.9–14.2)	8.4 (5.0–14.9)
Hospital mortality	1633 (17.8)	11,998 (8.6)	13,631 (9.1)

In total, 9179 patients (6.1%) developed an acute episode of delirium during ICU admission, and 4142 (2.8%) of the total patients were frail and experienced acute delirium during their ICU admission. Larger proportions of frail than non‐frail patients (45.1% vs 26.8%) developed delirium. Patients who developed delirium were more frequently admitted to ICU from emergency departments (31.0% vs 22.5%) or had longer hospital stay (median length of stay 14.1 vs 8.2 days) than patients without delirium.

### Association between frailty, delirium, and mortality

**Fig. 2:**
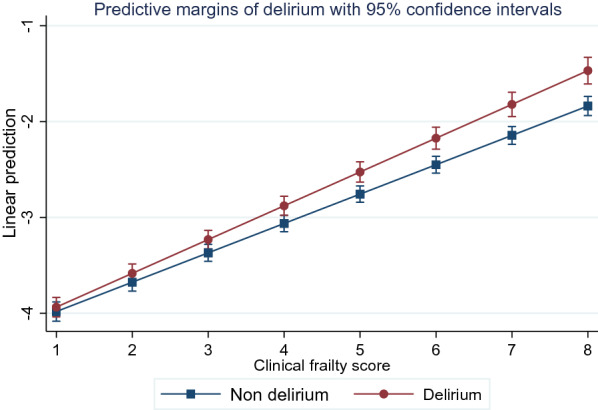
The interaction between clinical frailty score and delirium on in-hospital mortality

In the analysis adjusted for ANZROD, frail patients had significantly higher odds of in-hospital mortality (odds ratio [OR]: 2.15, 95% confidence interval [CI] 2.05–2.25) and an episode of delirium (OR: 1.86, 95% CI 1.77–1.95). After adjusting for ANZROD and frailty, acute delirium was associated with 32% higher odds of in-hospital mortality (OR: 1.32, 95% CI 1.23–1.43). The association between frailty and in-hospital mortality was modified by acute delirium (Fig. 2). Compared to non-frail patients without acute delirium, the odds ratios (95% CI) for in-hospital mortality were 1.37 (1.23–1.52), 2.14 (2.04–2.24) and 2.77 (2.51–3.05) for non-frail who developed delirium, frail without delirium, and frail who developed delirium during ICU admission, respectively (Table 2). Table 3 presents results of the mediation analysis on mortality. A very small but statistically significant effect of frailty on in-hospital mortality was mediated through an episode of delirium (*b* for indirect effect = 0.00037, *P* < 0.001). Overall, the indirect effect of frailty on mortality through an episode of acute delirium accounted for only 1.1% of the total effect of frailty on mortality.

**Table 2:** Association between frailty, delirium and in-hospital mortality and length of stay

	Risk of in-hospital mortality	*P*-value	Log transformed length of hospital stay	*P*-value
	Adjusted odds ratio (95% CI)		Mean difference (95% CI)	
Frailty				
No	Ref		Ref	
Yes	2.15 (2.05–2.25)	< 0.001	0.24 (0.23–0.25)	< 0.001
Episode of delirium#				
No	Ref		Ref	
Yes	1.32 (1.23–1.43)	< 0.001	0.52 (0.50–0.54)	< 0.001
Frailty predicting delirium				
Frailty				
No	Ref			
Yes	1.86 (1.77–1.95)	< 0.001		
Interaction between frailty and delirium				
Not frail- without delirium	Ref		Ref	
Not frail—with delirium	1.37 (1.23–1.52)	< 0.001	0.62 (0.60–0.65)	< 0.001
Frail—no delirium	2.14 (2.04–2.24)	< 0.001	0.24 (0.23–0.25)	< 0.001
Frail with delirium	2.77 (2.51–3.05)	< 0.001	0.62 (0.59–0.65)	< 0.001

Adjusted for Australian and New Zealand Risk of Death (ANZROD). ANZROD is derived from patient and clinical characteristics, including the Acute Physiology and Chronic Health Evaluation (APACHE) III, ICU admission source, admission diagnoses, Acute Physiology score (APS), APACHE III chronic health score, treatment limitation, and ventilation status

^#^Adjusted for ANZROD and frailty

**Table 3:** Association between frailty and in-hospital mortality and length of stay mediated by acute delirium

	In-hospital mortality	SE	Z	*P*-value
	b (95% CI)			
Direct effect (c′)	0.0330 (0.031–0.038)	0.002	20.11	< 0.001
Indirect effect	0.00037 (0.00008–0.0007)	0.00015	2.50	< 0.012
Total effect	0.0344 (0.0313–0.0381)	0.001	20.36	< 0.001
% of total effect mediated	1.1%			
% of total effect mediated^#^	1.8%			
	Hospital length of stay			
Direct effect (c′)	0.202 (0.19–0.21)	006	34.76	< 0.001
Indirect effect	0.019 (0.017–0.021)	0.001	20.96	< 0.001
Total effect	0.22 (0.21–0.23)	0.006	37.48	< 0.001
% of total effect mediated	8.6%			
% of total effect mediated^#^	9.3%			

Adjusted for Australian and New Zealand Risk of Death (ANZROD). ANZROD is derived from patient and clinical characteristics, including the Acute Physiology and Chronic Health Evaluation (APACHE) III, ICU admission source, admission diagnoses, Acute Physiology score (APS), APACHE III chronic health score, treatment limitation, and ventilation status

^#^Analysis adjusted for age and sex only

### Association between frailty, delirium, and length of stay

After adjusting for ANZROD, frailty (log-transformed mean difference (MD): 0.24, 95% CI 0.23–0.25) and an episode of delirium (*b* = 0.54, 95% CI 0.52–0.56) were associated with significantly longer LOS. The interaction between frailty and delirium on LOS was statistically significant. Non-frail patients who developed acute delirium (*MD* = 0.62, 95% CI 0.60–0.65) had three times longer LOS than frail patients without acute delirium (*MD* = 0.24, 95% CI 0.23–0.25) (Table 2). There was small (8.6%) but statistically significant effect of frailty on LOS mediated through acute delirium (b for indirect effect: 0.019, 95% CI 0.017–0.021) (Table 3). The associations were consistent when the frailty was modelled as a continuous variable. Higher frailty scores were associated with higher risk of delirium, mortality, and LOS (Additional file 2: Table S2).

## Discussion

In this large multicentre registry of critically ill patients, frailty was associated with increased risk of in-hospital mortality and longer LOS. Delirium is common in frail patients and is independently associated with increased risk of in-hospital mortality and longer LOS. We also found that the proportions of the effect of frailty on in-hospital mortality and LOS mediated or modified by delirium were statistically significant but very negligible.

The prevalence of frailty reported in our study is comparable with that reported by a large Canadian study of 15,238 critically ill adults (28%) [26], and by a meta-analysis of patients admitted to ICU (pooled prevalence of 30%) [3]. However, a higher frailty prevalence (30–46%) has also been reported by large cohort studies [10, 13, 27]. The Very Old Intensive Care Patients (VIP Study) found that 42.9% of patients aged ≥ 80 years old admitted to ICU were frail [10]. Although it is established that frailty is prevalent in critically ill adults, its prevalence varies by age, sex, case-mix, acuity of illness and frailty classification and assessment tools [18, 26].

The incidence of delirium in our study was considerably lower than those reported in previous studies. A prospective follow-up of 997 critically ill patients (mean age: 71 years) reported a higher episode of delirium (13%) [13]. Two large meta-analyses studies showed that the incidence of delirium in critically ill adults ranges from 16 to 31% [12, 28], although the incidence of delirium could be higher depending on the patient populations. The patients in our study had a short LOS (median: 8.4 vs 10 days), had a less severe illness (median APACHE III score: 54 vs 56), and a smaller proportion of patients had sepsis (8.9%) or chronic respiratory diseases (11.3%) than reported in other studies [13, 29].

Our findings of the higher risk of in-hospital death and longer LOS in patients who were frail at ICU admission are consistent with the literature [3, 13]. Sanchez et al. reported that, among adults aged 50 years or more, frailty at ICU admission was associated with increased risk of hospital mortality (OR: 2.54) and longer LOS (mean difference: 2.6 days) [13]. A meta-analysis of 3030 critically ill adults reported a pooled odds ratio for in-hospital mortality of 1.71 (95% CI 1.43–2.05), but non-statistically significant longer stays (3.39 days, 95% CI − 0.33 to 7.10) [3]. In our study, the increased risks of in-hospital mortality and longer LOS associated with frailty were independent of potential confounders, including severity of illness, chronic comorbidities and admission diagnosis suggesting the clinical and public health importance of frailty on its own.

Similarly, our findings of increased risk of in-hospital mortality and longer LOS associated with an episode of delirium are consistent with previous studies that reported significantly higher mortality and LOS in patients with delirium [12, 13]. Sanchez et al. found that compared to ICU patients without delirium, patients with delirium had significantly higher risk of hospital mortality (OR: 2.03) and longer LOS (mean difference: 2 days) [13]. A meta-analysis of 42 studies (16,595 critically ill patients) reported that patients with delirium had significantly higher hospital mortality (risk ratio: 2.19, 94% CI 1.78–2.70) as well as longer LOS (mean difference: 0.97 days, 95% CI 0.61–1.33) [12].

A key finding of this study is that acute delirium is more common in frail patients; however, it does not have a clinically meaningful influence on the poor prognosis in frail patients. The proportion of the effect of frailty on in-hospital mortality and LOS mediated through acute delirium were 1.1% and 8.6%, respectively, which are very small effect sizes [30]. The mediating role of delirium in the associations between frailty and in-hospital mortality and LOS is plausible. It is established that frailty independently increases the risk of delirium, [9, 14] which in turn increases the risk of adverse clinical outcomes [7, 12]. There are several reasons for the why the mediated effect sizes could be statistically significant but not a clinically meaningful. It has been suggested that measurement error in the mediator variable tends to suppress the mediated effect size, [31] which could be the case for delirium whose measurement poses unique challenges. Furthermore, the mediated effect size could decrease with increasing number of potential confounders included in the analyses [31]. However, the mediating effects in our study did not change when the analyses were adjusted for age and sex only. Larger studies like ours are also more likely to detect statistically significant mediation effects with small effect sizes that may not be clinically relevant.

Although acute delirium significantly modifies the association between frailty and in-hospital mortality and longer LOS, there was no clinically meaningful difference in the strength of the association between frailty and in-hospital mortality and LOS in those with and without acute delirium. Previous studies reported inconsistent results on whether and to what extent acute delirium modifies the association between frailty and in-hospital mortality or LOS. Sanchez et al. found that frail patients who developed acute episode of delirium (OR: 4.16, 1.50–11.52) had higher risk of hospital mortality than who did not (OR: 2.24, 95% CI 1.37–3.67) [13]. In another study of patients (≥ 70 years) admitted to acute medical care, the overall impact of delirium on admission tends to be greater at lower levels of frailty (*P* = 0.07) [15]. A study of 2,065 patients aged 65 years or older hospitalized in 118 acute medical wards and 46 rehabilitation units in Italy found that there was no interaction between delirium and frailty on 30-day mortality (*P* = 0.477) [32]. The discrepant findings between studies may relate in part to variations in study populations, sample size, and tools for the assessment of frailty and delirium [6, 33].

Although the degree to which delirium influence the effect of frailty on mortality outcomes and longer length of stay is very small, the higher risk of delirium in frail patients could have important clinical implications. Given that acute delirium is underrecognized and underdiagnosed, standardized assessment of frailty in ICU might facilitate identification of those at greater risk of acute delirium who could benefit from prevention, early recognition and evidence-based treatment.

This study has some limitations. Assessment of frailty and delirium in ICU settings poses many challenges, including lack of standardized, feasible and acceptable assessment tools and procedures. The absence of ICU level requirement or agreed process for assessing or diagnosing delirium in all patients could potentially lead to underdiagnosis of delirium patients. The ANZCIS registry uses CFS and has been assessed to be a valid measurement tool for frailty in the critically ill patients, compared with the multidimensional tool, and the Edmonton Frail Scale [34]. However, delirium was not further categorized into subtypes (hyperactive, hypoactive, mixed delirium) in our study although different subtypes of delirium are associated with differing outcomes. Treatments and therapies which might modify the detection, incidence or effect of delirium were not measured, therefore were not taken into account in the analyses. The type of tools used to diagnose delirium was not collected, and therefore we could not assess whether the association between frailty and delirium varies according to the tools used to diagnose delirium. Exclusion of patients who had delirium prior to ICU admission may contribute to lower rates of delirium. Furthermore, the incidence of delirium may have been underestimated because common tools such as CAM-ICU have limited sensitivity for detecting hypoactive delirium.

## Conclusions

In this large cohort of critically ill adults, frailty was associated with increased risk of in-hospital mortality, and longer LOS. Acute delirium was more common in frail patients and independently associated with in-hospital mortality and longer LOS. Acute delirium does not mediate or modify a clinically meaningful amount in-hospital mortality and LOS associated to frailty. Standardized screening and assessment of frailty as part of routine ICU care could improve not only early identification and management of older adults with frailty, but also early recognition of individuals at greater risk of delirium during ICU admission who would benefit from evidence-based interventions.

## Supplementary Information

**Additional file 1: **STROBE Statement—checklist of items that should be included in reports of observational studies.

**Additional file 2: Table S1.** Comparison of characteristics of patients who had data on frailty and delirium and those who did not. **Table S2.** Association between continuous frailty scores, delirium and in-hospital mortality and length of stay. **Table S3.** List of participating hospital in the study.

## Data Availability

The datasets used in the current study are not publicly available, but are available from the corresponding author on reasonable request.

## Abbreviations

ANZICS: Australia and New Zealand Intensive Care Society
ANZROD: Australia and New Zealand Risk of Death
APACHE: Acute Physiology and Chronic Health Evaluation
APD: Adult Patient Database
CFS: Clinical Frailty Scale
ICU: Intensive care unit
IQR: Interquartile range
LOS: Length of stay
MD: Mean difference
OR: Odds ratio
