# Supplementary material for: Association between frailty, delirium, and mortality in older critically ill patients: a binational registry study
Source: Ann Intensive Care. 2022 Nov 17;12:108. doi: 10.1186/s13613-022-01080-y (PMC9672151; doi:10.1186/s13613-022-01080-y)
Supplement: Supplementary file 2 — Additional file 2: Table S1. Comparison of characteristics of patients who had data on frailty and delirium and those who did not. Table S2. Association between continuous frailty scores, delirium and in-hospital mortality and length of stay. Table S3. List of participating hospital in the study. [file 13613_2022_1080_MOESM2_ESM.docx]

**Table S1: Comparison of characteristics of patients who had data on frailty and delirium and those who did not**

| **Characteristics** | **Excluded** | **Included** |
| --- | --- | --- |
| Number | 182,512 | 149,320 |
| Age (years), median (IQR) | 74.52 (69.5-80.44 | 75.2 (70.3-81.1) |
| Sex (men) | 108376 (59.4) | 86,153 (57.7) |
| **Admission diagnosis** |  |  |
| Cardiovascular disease | 53476 (29.3) | 40,726 (27.3) |
| Gastrointestinal | 32122 (17.6) | 26,935 (18.0) |
| Respiratory disease | 25187 (13.8) | 22,181 (14.8) |
| Sepsis | 14966 (8.2) | 13,352 (8.9) |
| Neurological disorders | 20806 (11.4) | 12,644 (8.5) |
| Chronic respiratory disease | 17521 (9.6) | 16,868 (11.3) |
| Cardiovascular disease | 16244 (8.9) | 23,493 (15.7) |
| Chronic renal failure | 6023 (3.3) | 7,362 (4.9) |
| Chronic liver disease | 2008 (1.1) | 1562 (1.0) |
| Metastatic cancer | 7300 (4.4) | 7,352 (4.9) |
| Hospital type |  |  |
| Tertiary | 69355 (38.0) | 41,016 (27.5) |
| Metropolitan | 24274 (13.3) | 25,585 (17.1) |
| Rural/regional | 25004 (13.7) | 25,208 (16.9) |
| Private | 63697 (34.9) | 57,511 (38.5) |
| APACHE III score, median (IQR), % | 54 (43-68) | 54 (43-67) |
| APACHE III predicted mortality, mean (SD), % | 15.6 (20.0) | 15.0 (19.0) |
| ANZROD, median (IQR), % | 2.3 (0.8-9.1) | 2.3 (0.7-9.1) |
| ANZROD, mean (SD), % | 9.8 (17.7) | 9.5 (17.1) |
| Length of hospital stay, median (IQR), d | 8.9 (5.2-15.6) | 8.4 (5.0-14.9) |
| Hospital mortality | 17156 (9.4) | 13,631 (9.1) |

APACHE- Acute Physiology and Chronic Health Evaluation; Australian and New Zealand Risk of Death (ANZROD) model; SD-standard deviation; ICU: Intensive Care Unit; IQR-interquartile range.

**Table S2: Association between continuous frailty scores, delirium and in-hospital mortality and length of stay**

| Frailty | Risk of in-hospital mortality  Adjusted OR (95% CI) | P-value |  |
| --- | --- | --- | --- |
| No | Ref |  |  |
| Yes | 1.37 (1.34-1.39) | <0.001 |  |
|  | Risk of delirium  Adjusted OR (95% CI) |  |  |
| Frailty |  |  |  |
| No | Ref |  |  |
| Yes | 1.26 (1.24-1.28) | <0.001 |  |
| Interaction between frailty and delirium | Risk of in-hospital mortality  Adjusted OR (95% CI) |  |  |
| Interaction between frailty and delirium |  |  |  |
| Not frail- without delirium | Ref |  |  |
| Not frail- with delirium | 1.36 (1.34-1.38) | <0.001 |  |
| Frail with delirium | 1.42 (1.39-1.45) | <0.001 |  |
| Frailty predicting Log transformed length of hospital stay | | | |
| Frailty | Log transformed length of hospital stay, β (95% CI) | P-value |  |
| No | Ref |  |  |
| Yes | 0.097 (0.094-0.100) | <0.001 |  |
| Interaction between frailty and delirium |  |  |  |
| Not frail- without delirium | Ref |  |  |
| Not frail- with delirium | 0.085 (0.081-0.089) | <0.001 |  |
| Frail with delirium | 0.176 (0.171-0.181) | <0.001 |  |

OR- Odds Ratio. β-regression coefficient. CI: Confidence Interval.

Adjusted for Australian and New Zealand Risk of Death (ANZROD). ANZROD is derived from patient and clinical characteristics, including the Acute Physiology and Chronic Health Evaluation (APACHE) III, ICU admission source, admission diagnoses, Acute Physiology score (APS), APACHE III chronic health score, treatment limitation, and ventilation status.

#Adjusted for ANZROD and frailty

**Table S3: List of participating hospital in the study**

Albury Base Hospital ICU; Alfred Hospital ICU; Alice Springs Hospital ICU; Allamanda Private Hospital ICU; Angliss Hospital ICU; Armadale Health Service ICU; Ashford Community Hospital ICU; Auckland City Hospital CV ICU; Auckland City Hospital DCCM; Austin Hospital ICU; Ballarat Health Services ICU; Bankstown-Lidcombe Hospital ICU; Bathurst Base Hospital ICU; Bendigo Health Care Group ICU; Blacktown Hospital ICU; Box Hill Hospital ICU; Brisbane Private Hospital ICU; Brisbane Waters Private Hospital ICU; Buderim Private Hospital ICU; Bunbury Regional Hospital ICU; Bundaberg Base Hospital ICU; Caboolture Hospital ICU; Cabrini Hospital ICU; Cairns Hospital ICU; Calvary Bruce Private Hospital HDU; Calvary Hospital (Canberra) ICU; Calvary Hospital (Lenah Valley) ICU; Calvary John James Hospital ICU; Calvary Mater Newcastle ICU; Calvary North Adelaide Hospital ICU; Calvary Wakefield Hospital (Adelaide) ICU; Campbelltown Hospital ICU; Canberra Hospital ICU; Casey Hospital ICU; Central Gippsland Health Service ICU; Christchurch Hospital ICU; Coffs Harbour Health Campus ICU; Concord Hospital (Sydney) ICU; Dandenong Hospital ICU; Dubbo Base Hospital ICU; Dunedin Hospital ICU; Epworth Eastern Private Hospital ICU; Epworth Freemasons Hospital ICU; Epworth Geelong ICU; Epworth Hospital (Richmond) ICU; Fairfield Hospital ICU; Figtree Private Hospital ICU; Fiona Stanley Hospital ICU; Flinders Medical Centre ICU; Flinders Private Hospital ICU; Footscray Hospital ICU; Frankston Hospital ICU; Fremantle Hospital ICU; Gold Coast Private Hospital ICU; Gold Coast University Hospital ICU; Gosford Hospital ICU; Gosford Private Hospital ICU; Goulburn Base Hospital ICU; Goulburn Valley Health ICU; Grafton Base Hospital ICU; Greenslopes Private Hospital ICU; Griffith Base Hospital ICU; Hawkes Bay Hospital ICU; Hervey Bay Hospital ICU; Hollywood Private Hospital ICU; Holmesglen Private Hospital ICU; Holy Spirit Northside Hospital ICU; Hornsby Ku-ring-gai Hospital ICU; Hurstville Private Hospital ICU; Hutt Hospital ICU; Ipswich Hospital ICU; John Fawkner Hospital ICU; John Flynn Private Hospital ICU; John Hunter Hospital ICU; Joondalup Health Campus ICU; Kareena Private Hospital ICU; Knox Private Hospital ICU; Latrobe Regional Hospital ICU; Launceston General Hospital ICU; Lingard Private Hospital ICU; Lismore Base Hospital ICU; Liverpool Hospital ICU; Logan Hospital ICU; Lyell McEwin Hospital ICU; Mackay Base Hospital ICU; Macquarie University Private Hospital ICU; Maitland Hospital HDU/CCU; Maitland Private Hospital; Manly Hospital & Community Health ICU; Manning Rural Referral Hospital ICU; Maroondah Hospital ICU; Mater Adults Hospital (Brisbane) ICU; Mater Health Services North Queensland ICU; Mater Private Hospital (Brisbane) ICU; Mater Private Hospital (Sydney) ICU; Melbourne Private Hospital ICU; Middlemore Hospital ICU; Mildura Base Hospital ICU; Modbury Public Hospital ICU; Monash Medical Centre-Clayton Campus ICU; Mount Hospital ICU; Mount Isa Hospital ICU; Nambour General Hospital ICU; National Capital Private Hospital ICU; Nelson Hospital ICU; Nepean Hospital ICU; Nepean Private Hospital ICU; Newcastle Private Hospital ICU; Noosa Hospital ICU; North Shore Hospital ICU; North Shore Private Hospital ICU; North West Regional Hospital (Burnie) ICU; Northeast Health Wangaratta ICU; Northern Beaches Hospital; Norwest Private Hospital ICU; Orange Base Hospital ICU; Peninsula Private Hospital ICU; Peter MacCallum Cancer Institute ICU; Pindara Private Hospital ICU; Port Macquarie Base Hospital ICU; Prince of Wales Hospital (Sydney) ICU; Prince of Wales Private Hospital (Sydney) ICU; Princess Alexandra Hospital ICU; Queen Elizabeth II Jubilee Hospital ICU; Redcliffe Hospital ICU; Repatriation General Hospital (Adelaide) ICU; Robina Hospital ICU; Rockhampton Hospital ICU; Rockingham General Hospital ICU; Rotorua Hospital ICU; Royal Adelaide Hospital ICU; Royal Brisbane and Women's Hospital ICU; Royal Darwin Hospital ICU; Royal Hobart Hospital ICU; Royal Melbourne Hospital ICU; Royal North Shore Hospital ICU; Royal Perth Hospital ICU; Royal Prince Alfred Hospital ICU; Ryde Hospital & Community Health Services ICU; Shoalhaven Hospital ICU; Sir Charles Gairdner Hospital ICU; South West Healthcare (Warrnambool) ICU; Southern Cross Hospital (Hamilton) ICU; Southern Cross Hospital (Wellington) ICU; St Andrew's Hospital (Adelaide) ICU; St Andrew's Hospital Toowoomba ICU; St Andrew's Private Hospital (Ipswich) ICU; St Andrew's War Memorial Hospital ICU; St George Hospital (Sydney) CICU; St George Hospital (Sydney) ICU; St George Hospital (Sydney) ICU2; St George Private Hospital (Sydney) ICU; St John of God (Berwick) ICU; St John Of God Health Care (Subiaco) ICU; St John Of God Hospital (Ballarat) ICU; St John of God Hospital (Bendigo) ICU; St John Of God Hospital (Geelong) ICU; St John Of God Hospital (Murdoch) ICU; St John of God Midland Public & Private ICU; St Vincent's Hospital (Melbourne) ICU; St Vincent's Hospital (Sydney) ICU; St Vincent's Hospital (Toowoomba) ICU; St Vincent's Private Hospital (Sydney) ICU; St Vincent's Private Hospital Fitzroy ICU; Sunnybank Hospital ICU; Sunshine Coast University Hospital ICU; Sunshine Coast University Private Hospital ICU; Sunshine Hospital ICU; Sutherland Hospital & Community Health Services ICU; Sydney Adventist Hospital ICU; Sydney Southwest Private Hospital ICU; Tamworth Base Hospital ICU; Taranaki Health ICU; Tauranga Hospital ICU; The Bays Hospital ICU; The Chris O’Brien Lifehouse ICU; The Memorial Hospital (Adelaide) ICU; The Northern Hospital ICU; The Prince Charles Hospital ICU; The Queen Elizabeth (Adelaide) ICU; The Townsville Hospital ICU; The Valley Private Hospital ICU; The Wesley Hospital ICU; Timaru Hospital ICU; Toowoomba Hospital ICU; Tweed Heads District Hospital ICU; University Hospital Geelong ICU; Wagga Wagga Base Hospital & District Health ICU; Waikato Hospital ICU; Warringal Private Hospital ICU; Werribee Mercy Hospital ICU; Western District Health Service (Hamilton) ICU; Western Hospital (SA) ICU; Western Private Hospital ICU; Westmead Hospital ICU; Westmead Private Hospital ICU; Whakatane Hospital ICU; Whangarei Area Hospital, Northland Health Ltd ICU; Wimmera Health Care Group (Horsham) ICU; Wollongong Hospital ICU; Wollongong Private Hospital ICU; Women's and Children's Hospital PICU; Wyong Hospital ICU
